# From Brain to Being: Reintegrating Philosophy Into Neurology Education

**DOI:** 10.1212/NE9.0000000000200245

**Published:** 2025-09-10

**Authors:** Eduardo Boiteux Uchôa Cavalcanti

**Affiliations:** 1 From the Neurology Outpatient Clinic, Rede SARAH de Hospitais de Reabilitação, Brasília, Brazil.

## Abstract

Neurologists increasingly face clinical situations marked by diagnostic ambiguity, ethical complexity, and disorders that challenge traditional concepts of consciousness, personhood, and agency. Yet most neurology training programs remain focused on biomedical knowledge and procedural skills, offering limited preparation for these profound and often morally charged aspects of care. This educational gap may undermine clinical reasoning, ethical sensitivity, and the formation of a reflective professional identity. This review proposes the integration of 3 underrepresented but essential domains—epistemology, ethics, and philosophy of mind—into neurology education. Guided by Kern's 6-step curriculum development model, the article outlines theory-informed, evidence-based strategies to embed philosophical competencies within postgraduate training. Epistemology supports diagnostic reasoning through metacognitive insight, recognition of bias, and tolerance for ambiguity. Ethics education strengthens moral judgment and communication in scenarios involving capacity assessment, end-of-life care, and neurotechnological interventions. Philosophy of mind offers conceptual clarity for understanding disorders of consciousness, neurodegeneration, and altered personhood. Curricular strategies include narrative debriefings, ethics Objective Structured Clinical Examinations (OSCEs), interdisciplinary seminars, and reflective bedside teaching, all of which can be embedded into core clinical rotations such as neuro-intensive care unit (ICU) and cognitive neurology. Learning objectives are aligned with Accreditation Council for Graduate Medical Education milestones and supported by validated assessment tools, including reflective writing rubrics, structured ethical evaluations, and measures of ambiguity tolerance. Implementation barriers—including faculty readiness and curricular constraints—are addressed through faculty development, co-teaching models, and modular integration. By reframing philosophy as a clinical competency rather than a theoretical enrichment, this review offers a pragmatic and forward-looking approach to neurology education. Embedding philosophical reasoning into training enhances diagnostic precision, ethical engagement, and patient-centered care. The integration of philosophy into neurology education is not a veneration of the past, but a forward-looking complement, offering a humanistic framework to guide clinical reasoning and professional identity in an era shaped by artificial intelligence and neurotechnology.

## Introduction

Neurology education is undergoing a pivotal transformation in response to rapid advances in neuroscience, artificial intelligence, and neurotechnology. Innovations such as brain–computer interfaces, algorithmic diagnostics, and precision biomarkers are redefining how neurologists acquire, interpret, and apply clinical knowledge.^1-3^ While these tools enhance diagnostic capabilities, they also introduce new layers of epistemic, ethical, and conceptual complexity that transcend procedural expertise.^2-6^

In parallel, medical education has increasingly shifted from memorization and technical proficiency toward the cultivation of clinical reasoning, moral judgment, and patient-centered.^7,8^ Neurology, particularly in the context of disorders of consciousness, functional neurologic symptoms, neurodegenerative diseases, and end-of-life care, offers a uniquely fertile ground for cultivating these competencies.^4,5,9,10^ However, many postgraduate programs continue to prioritize scientific knowledge and procedural training, often offering limited opportunities for structured reflection, ethical deliberation, or conceptual engagement.

Historically, medicine and philosophy were deeply intertwined. Foundational thinkers such as Hippocrates, Galen, and Avicenna treated philosophical inquiry as central to the ethical and epistemological dimensions of clinical practice.^11-13^ In contemporary education, this linkage has weakened. Despite growing recognition of the need for reflective and ethically grounded training, philosophical domains—epistemology, ethics, and philosophy of mind—remain underrepresented in neurology curricula.^14-17^

Several academic initiatives have begun to address this gap. Columbia University's Narrative Medicine, the *Philosophicum* at Würzburg, and Milan's FOLSATEC initiative exemplify structured attempts to incorporate reflection, narrative, and conceptual analysis into clinical education.^18-20^ Still, these efforts remain largely elective, fragmented, and inconsistently implemented. These models have demonstrated feasibility and perceived benefits, including increased empathy, tolerance for ambiguity, and identity formation.^18,19^ Nevertheless, many remain elective or peripheral, and formal evaluation data remain limited. Barriers such as time constraints, limited faculty expertise, and the influence of the hidden curriculum, which often valorizes decisiveness and procedural efficiency, continue to impede integration.^7,17,21^

This review argues that reintegrating philosophy into neurology education is both timely and necessary. Epistemology equips clinicians to navigate uncertainty, mitigate bias, and reflect on how knowledge is constructed.^8,21,22^ Ethics enables principled engagement with moral dilemmas, from surrogate decision-making to neurotechnological interventions.^4-6,16^ The philosophy of mind provides essential tools to interpret identity, agency, and consciousness, concepts central to many neurologic conditions.^9,23^

Using Kern's 6-step curriculum development model as an organizing framework, this article synthesizes evidence-informed strategies for embedding these domains into neurology training.^24^ It also addresses institutional and pedagogical barriers to implementation and proposes realistic solutions. Rather than offering philosophy as an enrichment or add-on, this review positions it as a core competency for the modern neurologist.

## Epistemology and Diagnostic Reasoning

Diagnostic uncertainty is a defining feature of neurology. Presentations are often subtle, evolving, or resist categorical classification, challenging clinicians to make nuanced judgments amid incomplete or ambiguous information. In such contexts, diagnostic accuracy depends not only on factual knowledge but also on epistemological competence, an understanding of how knowledge is constructed, interpreted, and applied under conditions of uncertainty.^8,21^

Despite this, epistemology remains underrepresented in neurology training. Cognitive biases such as anchoring, premature closure, and attribution errors are well-documented contributors to diagnostic failure, yet most training emphasizes knowledge acquisition over metacognitive awareness.^21,25,26^ Educational interventions tend to emphasize knowledge acquisition rather than cultivating the metacognitive awareness needed to recognize and correct faulty reasoning.^8,21,25^ As a result, many trainees lack structured opportunities to reflect on their reasoning processes or to recognize the limitations of their conclusions.^25,26^

Epistemological training directly addresses these limitations by developing what Veen and Cianciolo^17^ describe as the ability to operate in the “muddy zone” of clinical reasoning, where algorithmic closure is not possible and interpretive judgment is essential. This capacity is especially relevant in neurology, where contested diagnoses, uncertain pathophysiology, and context-sensitive presentations are common.

Beyond dual-process models of cognition, contemporary frameworks encourage epistemic pluralism: the integration of empirical evidence with narrative reasoning, patient context, and experiential insight.^21,22,27^ Tonelli and others argue that such pluralism is essential for navigating complexity in a way that respects the limitations of purely data-driven approaches.^21^

A foundational construct within this pluralistic view is epistemic humility—the recognition of one's cognitive limits, willingness to revise beliefs, and openness to alternative interpretations.^21,22^ Targeted training in metacognition, as shown by Vaid et al.,^28^ has been found to improve clinicians' tolerance for uncertainty and supports adaptive decision-making. Fricker^29^'s notion of epistemic injustice, the systematic devaluation of certain voices or sources of knowledge, further highlights the ethical imperative of inclusive, reflexive clinical reasoning that attends not only to what is known but also to whose knowledge is considered credible.

Table 1 outlines structured strategies for cultivating epistemic competence. These interventions can be embedded within bedside teaching, diagnostic rounds, and debriefings. Assessment tools include the Tolerance for Ambiguity Scale, reflective rubrics, and structured OSCEs designed to evaluate reasoning in ambiguous scenarios.^30-33^

**Table 1:** Educational Strategies to Foster Epistemic Competence in Neurology

Strategy	Educational goal	Learning context
Uncertainty rounds	Explore diagnostic ambiguity collaboratively	Morning reports, case discussions
Metacognitive workshops	Identify and analyze reasoning patterns and biases	Small-group teaching sessions
Narrative reflection	Examine interpretive assumptions and subjectivity	Reflective writing seminars
Simulated diagnostic cases	Assess adaptability and transparency in reasoning	Simulation centers, OSCEs

Implementation, however, encounters several challenges. Faculty often lack formal training in metacognition, diagnostic uncertainty, and cognitive bias; while prevailing institutional norms tend to prioritize decisiveness over critical reflection. Targeted faculty development programmes addressing these areas are therefore essential.^21,34,35^ In parallel, the hidden curriculum may implicitly discourage open engagement with doubt and ambiguity, thereby constraining opportunities for reflective inquiry.^36^

Although epistemological exposure may begin during undergraduate modules (e.g., “Mind, Brain, and Behavior”), neurology offers a uniquely fertile context for its expansion. Conditions such as disorders of consciousness and functional neurologic disorders compel clinicians to question diagnostic norms, navigate conceptual ambiguity, and communicate uncertainty to patients and families.^37-39^

By embedding epistemology into clinical reasoning curricula, programs can help trainees move beyond certainty-driven heuristics toward a more reflective, pluralistic, and ethically responsive form of diagnostic practice. This is not only educationally valuable but professionally necessary in a specialty that so often operates at the boundaries of knowledge.

## Ethics and Moral Judgment

Ethical reasoning is a core competency in neurology. Clinicians frequently face dilemmas involving surrogate decision-making, capacity assessment, end-of-life care, and emerging neurotechnologies.^4,16,40^ These situations require more than technical skill, they demand moral sensitivity, principled deliberation, and value-informed communication.^4,5,10,16,41^

Although ethical dilemmas are both frequent and consequential in neurologic practice, ethics education remains inconsistently structured and often marginalized within residency curricula.^42,43^ Surveys reveal that many residents feel inadequately prepared to navigate ethically complex situations, with substantial variability observed across programmes.^43,44^ The gaps in ethical training may compromise clinicians' ability to manage prognostic uncertainty, communicate with surrogate decision-makers, and balance autonomy and beneficence in the care of cognitively impaired patients.^16,45^

Both the American Academy of Neurology and Accreditation Council for Graduate Medical Education (ACGME) recognize ethics as a core domain of professional development.^41,46^ However, opportunities for guided reflection, skills-based training, and structured feedback are often limited. The hidden curriculum, the informal institutional norms that reward procedural efficiency or hierarchical deference, may further inhibit ethical reflection and discourage moral inquiry.^16,42,45,47^

Evidence-based instructional methods can bridge this gap. Table 2 outlines structured strategies for ethics education tailored to neurology. These strategies have shown positive effects on moral awareness, empathy, and professionalism.^42,44,48^ Simulation-based learning and narrative reflection, in particular, enable learners to explore ambiguity and emotional nuance in ways that standard lectures may not.^48,49^ Assessment tools, including ethics-focused OSCEs, moral distress scales, and reflective writing rubrics, provide feasible mechanisms for evaluating ethical development longitudinally.^6,48^

**Table 2:** Structured Methods for Teaching Ethical Reasoning in Neurologic Practice

Strategy	Educational goal	Learning context
Case-based seminars	Apply ethical reasoning to real-world neurologic dilemmas	Ethics rounds, journal clubs
Simulation exercises	Practice ethically fraught interactions (e.g., capacity, end-of-life)	Simulation labs, OSCEs
Narrative reflection	Deepen moral imagination and empathy	Reflective writing, story-based seminars

The rapid emergence of neurotechnologies further intensifies ethical complexity.^50^ Brain–computer interfaces, AI-assisted diagnostics, and cognitive enhancement tools challenge traditional ethical frameworks regarding autonomy, consent, mental privacy, and distributive justice.^1,2,51,52^ In response, the concept of “neurorights” has gained visibility, advocating legal and ethical protections for cognitive liberty, personal identity, mental integrity, and psychological continuity.^2,51,53,54^

Neurorights represent a potentially valuable framework for addressing emerging neuroethical issues. However, their integration into educational curricula remains challenging. There are ongoing debates about their conceptual clarity; their overlap with existing rights such as autonomy, privacy, and informed consent; and the impact of cultural differences in ethical norms.^40,55^ These discussions should be used as opportunities for critical thinking and analysis in ethics education. At the same time, educational programs should highlight the importance of promoting global equity in the development, regulation, and governance of neurotechnologies.^16,43^

Embedding ethics education into neurology training fosters clinicians capable of navigating these frontiers proactively and responsibly. Integration should occur not only through electives or ad hoc sessions but within core clinical experiences such as palliative care rounds, neuro-ICU debriefings, and interdisciplinary ethics panels.^48^ These settings lend authenticity and allow learners to link abstract ethical frameworks with lived clinical dilemmas.

Implementation barriers include faculty discomfort with leading ethical discussions, lack of protected teaching time, and the influence of hidden curriculum dynamics.^36,56^ Mitigation strategies include faculty development in ethics facilitation and values-based communication; integrating ethics into core clinical rotations rather than limiting it to electives; and partnering with humanities scholars, chaplains, or ethicists to expand teaching capacity.

Framing ethics as central to diagnostic and prognostic reasoning rather than as an ancillary or compliance topic can increase institutional engagement. Ethics education should be presented as essential to high-quality clinical care, identity formation, and the cultivation of moral resilience.

Looking forward, outcome research is needed to assess the impact of ethics curricula on clinician behavior, communication skills, and patient outcomes. Prospective evaluations, structured reflections, and qualitative assessments of learner narratives may help build the evidence base. Cultivating ethical reasoning within neurology ensures that trainees are not only clinically adept but also morally attuned to the complexities of neurologic care.

## Philosophy of Mind and Personhood

Neurologists frequently confront clinical situations that challenge conventional understandings of consciousness, identity, and agency.^5,9,23^ Conditions such as disorders of consciousness, neurodegenerative diseases, and neuropsychiatric syndromes prompt foundational questions: What constitutes awareness? What defines identity? How do we assess agency when memory or language is impaired? These questions are not merely theoretical; they have direct implications for prognostic judgment, therapeutic goals, and communication with patients and families.^4,5,9,10^

Despite their clinical relevance, most neurology training programs offer limited structured exposure to philosophy of mind. In practice, clinicians often rely on intuitive or culturally inherited assumptions, which may be inconsistent, reductionist, or ethically inadequate when facing conditions such as cognitive-motor dissociation or prolonged unresponsiveness.^23,37^

Philosophical frameworks offer interpretive tools for these challenges. Cognitive theories such as global workspace theory and higher-order thought models offer ways to conceptualize residual cognition in the absence of overt behavior.^57^ Integrated information theory provides a quantitative approach to consciousness, while enactivist and phenomenological models emphasize embodied and relational aspects of awareness. Narrative and relational theories of personhood shift emphasis from cognition alone to memory, moral agency, and social embeddedness.^9,20,23,58^

These perspectives are not merely theoretical, they shape how neurologists interpret behavior, set care goals, and counsel families. For instance, understanding personhood as relational rather than solely cognitive can support decisions that affirm dignity, connection, and meaning, even amid profound impairment. This reconceptualization fosters humility, empathy, and ethical coherence in treatment decisions.^7,16,18^

Several educational programs have piloted the integration of philosophy of mind. Initiatives such as Columbia University's Narrative Medicine and the *Philosophicum* at Würzburg report increased empathy, ambiguity tolerance, and reflective capacity among trainees.^18-20^ These outcomes suggest that philosophical instruction can enrich both cognitive and affective domains of medical education.

Table 3 outlines feasible pedagogical strategies for engaging learners with concepts of mind and personhood. These strategies should be embedded into clinical practice, not offered as isolated electives. When philosophical content is disconnected from patient care, learners may perceive it as irrelevant or overly abstract. To address this, programs should integrate conceptual reflection into existing venues—ethics consults, ICU debriefings, or cognitive rounds—where its relevance is immediate and authentic.

**Table 3:** Pedagogical Strategies to Explore Personhood and Philosophy of Mind

Strategy	Educational goal	Learning context
Narrative case analysis	Explore patient and caregiver experiences of altered personhood	Clinical debriefings, ethics seminars
Interdisciplinary seminars	Facilitate conceptual reflection with diverse experts	Grand rounds, humanities teaching
Rotational modules	Contextualize philosophical concepts in patient care	Neuro-ICU, cognitive neurology units
Conceptual debriefings	Examine assumptions after complex or ambiguous cases	Team meetings, bedside rounds

Faculty development is essential. Many clinicians may lack familiarity with philosophical discourse or feel unprepared to lead such discussions. Collaboration with experts in palliative care, chaplaincy, and the medical humanities can enrich instructional capacity and model interdisciplinary engagement.

Assessment tools such as empathy scales, DOC-focused OSCEs, and thematic analysis of reflective writing can provide formative insights into learners' conceptual and relational development.^9^ These instruments also align with broader educational goals related to professionalism, communication, and patient-centered care.

While foundational concepts may be introduced during undergraduate studies, neurology offers a uniquely rich context for deeper engagement. Neurologists often care for patients whose agency, moral status, or identity is uncertain or evolving.^9,16,23^ These situations require not only scientific reasoning but also conceptual and ethical reflection.

Philosophy of mind is therefore essential for addressing the interpretive and moral challenges posed by neurologic illness. When embedded in contextually grounded, interdisciplinary teaching, this content supports diagnostic sophistication, ethical discernment, and humanistic practice. Trainees gain tools not only to assess neurologic function but to care for persons in their full existential depth.

## Educational Integration and Curricular Strategy

The integration of philosophical competencies—epistemology, ethics, and philosophy of mind—into neurology education requires a deliberate, structured, and outcome-oriented approach. Kern's 6-step curriculum development model provides a practical guide for identifying educational gaps, aligning objectives with learned needs, designing interventions, and implementing and evaluating curricular change.^24^

## Step 1: Problem Identification and General Needs Assessment

Modern neurology demands interpretive judgment, ethical discernment, and nuanced understandings of consciousness and personhood.^7,9,16^ Yet most residency programmes emphasize technical and scientific proficiency while offering limited support for navigating ambiguity, conceptual complexity, or moral dilemmas. Trainees frequently report discomfort in managing uncertainty, conducting end-of-life discussions, or reasoning through disorders of consciousness.^5,35,48,59^ The absence of structured guidance in navigating these challenges can negatively impact the quality of patient care and may contribute to professional distress and burnout.^5,15,16,60^

## Step 2: Needs Assessment of Targeted Learners

Effective integration begins with a clear understanding of learners' challenges and context. Surveys and qualitative studies indicate that residents seek more structured guidance in ethical reasoning, uncertainty tolerance, and patient-centered reflection.^15-17,60^ These findings support a targeted curricular response that addresses epistemic, ethical, and conceptual reasoning as core competencies.

## Step 3: Goals and Objectives

The overarching goal is to cultivate reflective, ethically attuned, and conceptually literate neurologists. Learning objectives should be mapped to observable behaviors and aligned with competency-based assessment frameworks such as the ACGME Milestones (Table 4).^46^

**Table 4:** Measurable Learning Objectives and Corresponding Observable Behaviors

Domain	Objective (Bloom's Taxonomy)
Epistemology	Define key epistemic concepts (e.g., uncertainty, pluralism, bias); apply metacognitive strategies to diagnostic reasoning
Ethics	Analyze neurology-specific cases using ethical frameworks; demonstrate ethical communication in simulated interactions
Philosophy of Mind	Explain major theories of consciousness and personhood; reflect on assumptions regarding agency and identity in clinical scenarios

These objectives can be evaluated through reflective writing rubrics, ethics-focused OSCEs, and thematic analysis of learner responses to complex cases.

## Step 4: Educational Strategies

Integration should prioritize minimal curricular disruption by embedding philosophical content into existing structures. Modular, interdisciplinary, and case-based approaches are most effective (Table 5).

**Table 5:** Instructional Formats and Integration Sites for Philosophical Content

Format	Purpose	Integration site
Case conferences	Apply ethical and epistemic reasoning to clinical dilemmas	Morning reports, diagnostic rounds
Narrative debriefings	Reflect on interpretive and moral complexity	Bedside teaching, ethics consults
Interdisciplinary panels	Explore philosophical frameworks collaboratively	Grand rounds, CME events
Electives/Journal clubs	Deepen engagement with primary texts or cases	Optional modules, asynchronous learning

Additional strategies include quarterly theme-based seminars, flipped classroom formats, and philosophical touchpoints during high-yield rotations (e.g., neuro-ICU, cognitive neurology).

Mitigation strategies in neurology education include faculty development through focused workshops on cognitive bias, ethical reasoning, and conceptual facilitation. Time constraints can be addressed by integrating brief reflective elements into existing teaching sessions. To reduce cultural resistance, philosophy should be presented as a practical tool for improving diagnostic accuracy, ethical clarity, and professional identity.

## Step 5: Implementation

A phased approach is recommended. A pilot curriculum could be launched across 2 clinical rotations—such as neuro-ICU and cognitive neurology—with monthly philosophical integration sessions. Teaching could be co-facilitated by neurology faculty and external experts (e.g., ethicists, philosophers, chaplains).

Supplementary materials, including podcasts, reflective writing prompts, or short conceptual readings, can support asynchronous learning. Teaching sessions may be scheduled during academic half-days or integrated into postround debriefings.

Faculty recruitment should prioritize interprofessional diversity and include clinicians who model reflection, humility, and relational care. Evaluation metrics should be defined prior to launch and aligned with institutional objectives.

## Step 6: Evaluation and Feedback

Multimodal assessment ensures alignment with curricular goals and continuous improvement (Table 6).

**Table 6:** Outcome Measures and Assessment Tools for Evaluating Philosophical Competencies

Outcome	Assessment tool
Epistemic reflection and bias	Tolerance for Ambiguity Scale, structured reflective rubric
Ethical reasoning	Ethics OSCE stations, simulated decision-making
Conceptual engagement with personhood	Thematic analysis of reflective writing, DOC-focused OSCEs
Learner satisfaction	Post-session surveys, semi-structured interviews

Longitudinal evaluation may include documented participation in ethics consults, performance on milestone assessments related to professionalism and systems-based practice, or scholarly output (e.g., essays, case reflections). Results should inform ongoing curricular refinement and contribute to broader educational research.

Ultimately, curricular success should be measured not only by content delivery, but by its impact on clinical reasoning, moral integrity, and patient-centered care. When philosophy is presented as integral to the neurologist's identity—as thinker, moral agent, and interpreter of meaning—learners are more likely to engage with conceptual complexity as a central element of their professional development.

## Ensuring Balance and Relevance

While the integration of philosophical competencies into neurology education is pedagogically and ethically justified, it must be designed with precision and contextual relevance.^17,48,60^ Poorly structured initiatives, including isolated seminars or abstract theoretical modules, risk reinforcing the perception that philosophy is peripheral, impractical, or intellectually detached from clinical care.

Learner engagement may decline if content is not clearly mapped to clinical decision-making or patient outcomes. Similarly, philosophical instruction that displaces core biomedical content or overwhelms already dense curricula can trigger institutional resistance. To succeed, integration must be coherent, efficient, and visibly linked to existing training objectives.

Strategies to ensure balance include embedding philosophical reflection into established clinical structures—case conferences, ethics consults, neuro-ICU debriefing, where relevance is immediate and authentic. Instruction should be aligned with educational milestones and clearly mapped to observable behaviors. Interdisciplinary co-teaching with professionals in ethics, palliative care, chaplaincy, or the humanities enriches dialogue and broadens faculty capacity Figure.

**Figure:**
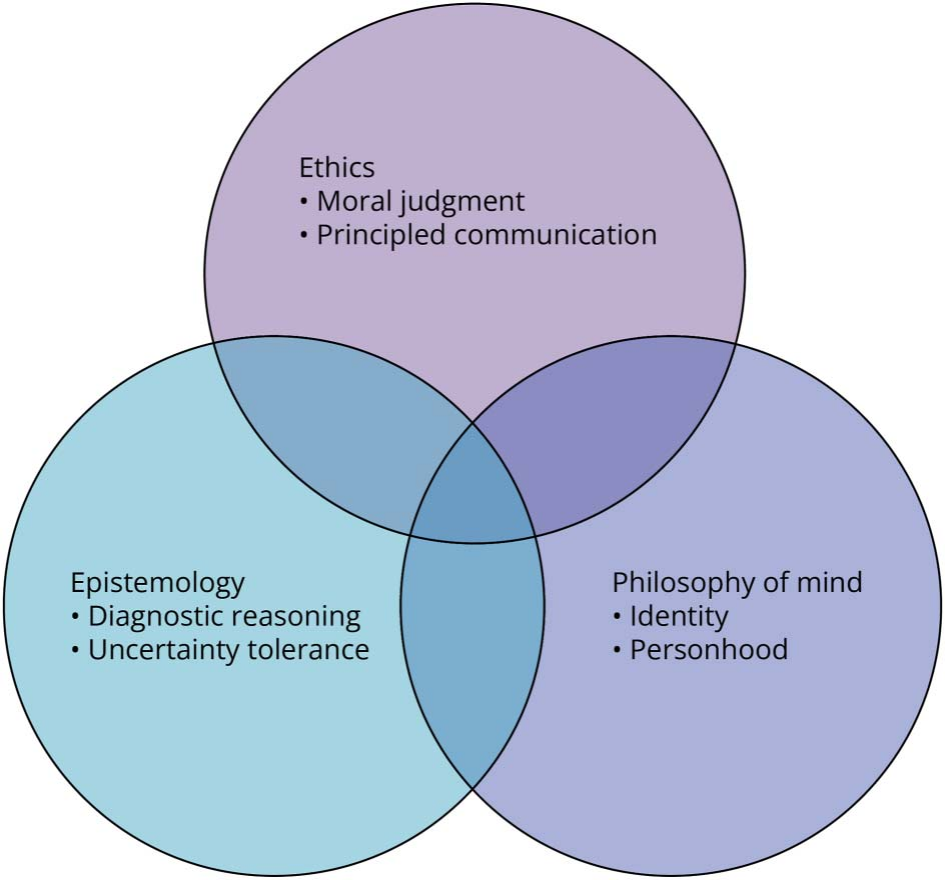
Key Domains of Philosophical Competence in Neurology The figure presents 3 domains—epistemology, ethics, and philosophy of mind—central to neurology training. Epistemology supports diagnostic reasoning and management of uncertainty; ethics informs clinical decision-making and professional conduct; philosophy of mind provides frameworks for understanding consciousness and personhood. These domains strengthen reflective and patient-centered neurologic practice.

Importantly, philosophy should not be framed as an academic digression or intellectual enrichment activity. Rather, it must be presented as a practical clinical tool essential for reasoning through diagnostic uncertainty, navigating moral complexity, and sustaining person-centered care. When aligned with institutional structures, supported by trained facilitators, and delivered through evidence-based pedagogy, the integration of philosophical competencies holds the potential to transform not only neurology education but also the identity and practice of the modern neurologist.

## Conclusion

Neurology education must prepare physicians not only to master scientific knowledge and procedural skills but also to reason through diagnostic ambiguity, address ethical dilemmas, and care for patients whose identities and capacities are shaped by neurologic illness. These challenges require more than technical acumen, they demand competencies that sustain clinical judgment, ethical sensitivity, and relational care.

This review has identified 3 foundational yet underrepresented domains—epistemology, ethics, and philosophy of mind—that are essential to contemporary neurology practice. When integrated through structured, context-sensitive strategies they enhance diagnostic reasoning and moral judgment and promote professional identity formation.

Using Kern's 6-step curriculum model, we propose a practical and scalable framework for embedding philosophical content into neurology education, aligned with existing competency-based standards.

As neurology evolves within a landscape of increasing moral complexity and rapid advancements in artificial intelligence, neurotechnology, and data-driven diagnostics, neurologists must be more than technicians. Instead, they must be thinkers, moral agents, and reflective practitioners, capable of exercising clinical judgment in an era increasingly mediated by algorithms and data automation.

Reintegrating philosophy into neurology education is not a nostalgic return to antiquity, but a deliberate step toward cultivating physicians equipped to lead with wisdom, communicate with integrity, and provide care grounded in humanistic values. The physician-philosopher is not a relic of the past. It is a model for the neurologist of the future.

## Glossary

ACGME: accreditation council for graduate medical education
